# Linguistic indicators for predicting the veracity of online health rumors

**DOI:** 10.3389/fpubh.2023.1278503

**Published:** 2024-01-10

**Authors:** Jingyi Zhao, Cun Fu

**Affiliations:** ^1^College of International Studies, Southwest University, Chongqing, China; ^2^School of Foreign Languages and Cultures, Chongqing University, Chongqing, China

**Keywords:** linguistic indicator, health rumors, veracity, logistic regression, prediction

## Abstract

This study aims to examine the role of language in discerning the authenticity of online health rumors. To achieve this goal, it specifically focuses on analyzing five categories of linguistic indicators: (1) emotional language characterized by sentiment words, sensory words, and continuous punctuations, (2) exaggerated language defined by the presence of extreme numbers and extreme adverbs, (3) personalized language denoted by first-person pronouns, (4) unprofessional language represented by typographical errors, and (5) linkage language marked by inclusion of hyperlinks. To conduct the investigation, a dataset consisting of 1,500 information items was utilized. The dataset exhibited a distribution pattern wherein 20% of the information was verified to be true, while the remaining 80% was categorized as rumors. These items were sourced from two prominent rumor-clarification websites in China. A binomial logistic regression was used for data analysis to determine whether the language used in an online health rumor could predict its authenticity. The results of the analysis showed that the presence of sentiment words, continuous punctuation marks, extreme numbers and adverbs in an online health rumor could predict its authenticity. Personalized language, typographical errors, and hyperlinks were also found to be useful indicators for identifying health rumors using linguistic indicators. These results provide valuable insights for identifying health rumors using language-based features and could help individuals and organizations better understand the credibility of online health information.

## Introduction

Health rumors are unverified or unconfirmed information related to health issues that spread among individuals or groups within a given community (1–3). They have the potential to create serious public health consequences, evoking widespread fear and anxiety, leading to stigmatization and promoting vaccine hesitancy or refusals. Therefore, it is crucial to address health rumors in order to promote accurate information and minimize the negative impact of outbreaks on affected communities.

Many researchers have explored the approach of detecting rumors with linguistic features. Some utilized automated linguistic analysis tools to identify linguistic patterns that are associated with rumors, such as the use of emotive language, vague references, and unverified claims. For example, Liu et al. (4) manually selected 104 linguistic and statistical features that are deemed useful for machine learning classifiers in detecting unreliable health-related information on Chinese social media.

By analyzing large amounts of text data from social media and other sources, some identified patterns that are indicative of rumors and distinguish them from factual information. For instance, Newman et al. (5) proposed a different approach that focuses on specific linguistic dimensions such as dictionary words, pronouns, and prepositions. Zhou et al. (6) categorized linguistic characteristics in a similar way to Burgoon et al. (7), but with more specific categories related to quantity, complexity, uncertainty, non-immediacy, expressivity, specificity, and affect. Zhang et al. (8) investigated the predictors of the authenticity of Internet health rumors. They found that the use of emotive language, first-person pronouns, and the presence of external links were significant predictors of the authenticity of health rumors on the Internet.

The detection of rumors has garnered substantial scrutiny from numerous scholars and various organizations. Despite the existence of certain fact-checking platforms like FactCheck and PolitiFact, effectively addressing the issue of identifying rumors remains an ongoing challenge. Moreover, considering the deleterious ramifications associated with health rumors, there exists a compelling scholarly imperative to undertake further investigations elucidating the linguistic characteristics intrinsic to health rumor discourse. Thus, this study aims to cultivate linguistic indicators of health rumors in Chinese context in terms of emotional language, exaggerated language, personalized language, unprofessional language and linkage language.

## Literature review

Studies of the linguistic features of rumors can date back to the studies of linguistic indicators of deceptive messages (9). In 1969, Ekman and Friesen published the first compelling theoretical statement on cues to deception, introducing the concept of two broad categories of cues: leakage cues and deception cues (10). Leakage cues can be generally viewed as non-verbal cues, while deception cues can be roughly considered as verbal cues. McCornack et al. (11) proposed that deceptive messages violated maxims of quantity, quality, manner and relation under the framework of information manipulation theory. Grice’s maxim of quantity pertains to the extent or magnitude of information conveyed during the process of communication. A violation of this maxim implies a lack of cooperative behavior on the part of the speaker by providing an inadequate amount of information. Deceptive messages violating this maxim demonstrate lower vocabulary complexity, grammar complexity, specificity and expressiveness. The violation of the quality maxim in communication occurs when the information provided by the speaker is false, unsupported, or lacks evidence. Deceptive messages violating this maxim contain more uncertainty than true ones. The violation of the manner maxim in communication occurs when the speaker’s message is unclear, ambiguous, or unnecessarily complex. Deceptive messages violating this maxim demonstrate more informality and non-immediacy. The violation of the relation maxim in communication occurs when the speaker’s message is irrelevant or unrelated to the ongoing conversation or topic. Deceptive messages violating this maxim contain redundancy words than truthful ones. The extant deception researches explored various linguistic indicators with the dimensions of above four maxims. Table 1 summarizes the linguistic indicators of deceptive messages.

**Table 1:** Linguistic indicators of deceptive messages.

Violation of maxims	Dimensions	Linguistic indicators	Findings	Sources
Violation of quantity	Vocabulary complexity	Word diversity	less	(6, 7, 12–14)
		Average word length	shorter	
	Grammatical complexity	Average sentence length	shorter	(6, 7, 21, 22)
		Average number of words	fewer	(7, 10, 13, 22)
			more	(6, 12, 14, 20)
		Average number of sentences	fewer	(21, 22)
			more	(20)
	Specificity	Number (e.g., 2, 8%, second)	fewer	(10, 14, 15)
		Modifiers (adjectives and adverbs)	fewer	(14, 15)
		Exclusion words (e.g., but, except, unless)	fewer	(23)
	Expressiveness	Sensory words (e.g., bright, soft, fragrant)	fewer	(15–17)
			more	(14)
		Sentiment words (e.g., exited, sad, angry)	more	(6, 7, 12–14, 20)
		Punctuation marks	more	(20)
Violation of quality	Uncertainty	Modal verbs (e.g., may, could, might)	more	(6, 13)
		qualifiers (e.g., maybe, perhaps, somewhat)	more	(13, 24)
Violation of manner	Informality	Typographical errors	more	(6, 20)
	Non-immediacy	First-person pronoun (e.g., I, me, myself)	less	(12, 14, 20, 23, 24)
			more	(17)
		Group reference (e.g., we, you, they)	more	(12, 13)
Violation of relation	irrelevant words	Redundancy (repetition of words or sentences)	more	(6, 20)

Deception studies reached some consensus concerning linguistic dimensions of deceptive languages. For instance, deceptive messages generally contained lower vocabulary complexity, simpler grammatical complexity, less specific and less expressive, more uncertainty, more informality and more redundancy but more sentiment words (7, 12–14). Nevertheless, findings on certain linguistic indicators are contradictory including the average number of words, average number of sentences, sensory words and first-person pronoun. For instance, some studies found that deceptive messages contained less sensory words such as bright, soft, and fragrant (15–17). Nevertheless, Hancock et al. (14) demonstrated that deceptive messages contained more sensory words.

Despite the extensive body of research investigating linguistic indicators of deception, relatively scanty studies have focused on this aspect within the context of health rumors. Table 2 illustrates the linguistic indicators investigated in extant health rumor studies.

**Table 2:** Linguistic indicators of health rumors.

Violation of maxims	Dimensions	Linguistic indicators	Findings	Sources
Violation of quantity	Vocabulary complexity	Word diversity	less	(8, 20, 25)
	Grammatical complexity	Average sentence length	shorter	(8, 25, 26)
		Average number of words	fewer	(25)
			more	(4, 8)
		Average number of sentences	fewer	(25)
			more	(4, 8)
	Specificity	Number (e.g., 2, 8%, second)	fewer	(4, 8, 20)
		Place names	fewer	(8, 25)
		Hyperlinks	fewer	(8, 25)
		Picture	fewer	(8)
		Time	fewer	(25)
		Exclusion words (e.g., but, except, unless)	fewer	(6, 27)
		Sentiment words (e.g., exited, sad, angry)	more	(25, 28)
			fewer	(29)
		Punctuation marks	more	(4)
Violation of quality	Uncertainty	Modal verbs (e.g., may, could, might)	more	(8, 9, 30)
		Qualifiers (e.g., maybe, perhaps, somewhat)	more	(8)
Violation of manner	Informality	Typographical errors	more	(8)
	Non-immediacy	First-person pronoun (e.g., I, me, myself)	less	(8, 9)
			more	(17)
Violation of relation	Irrelevant words	Redundancy (repetition of words or sentences)	more	(25)

Health rumor studies also obtain common findings on linguistic indicators including less word diversity, fewer number, more modal verbs, more typographical errors and more redundancy words. However, findings on average number of words, average number of sentences, sentiment words and first-person pronoun are inconsistent among these studies. For example, two studies illustrated that health rumors contain less first-person pronoun (8, 9), one study reached an opposite conclusion (18). In addition, findings on linguistic indicators such as sensory words, punctuation marks, typographical errors deserve further investigations. For instance, Although Zhang et al. (8) found that health rumors contained more typographical errors, Sitaula et al. (19)demonstrated that rumors obtain higher readability in terms of less typographical errors.

Thus, our study intends to investigate on some controversial linguistic indicators such as sensory words, first-person pronoun while further investigate linguistic indicators such as sentiment words, continuous punctuations, extreme numbers, extreme adverbs, typographical errors and hyperlinks. The above linguistic indicators are conceptualized into emotional language (sentiment words, sensory words, continuous punctuations), exaggerated language (extreme numbers, extreme adverbs), personalized language (first-person pronoun), unprofessional language (typographical errors) and linkage language (hyperlinks). Under the framework of information manipulation theory, emotional language, exaggerated language and linkage language violate the maxim of quantity while personalized language and unprofessional language violate the maxim of manner. Consequently, our study forms the following hypotheses:

*H*1: Information with emotional language (sentiment words, sensory words and continuous punctuations) are likely to be rumors.

*H*2: Information with exaggerated language (extreme number, extreme adverbs) are likely to be rumors.

*H*3: Information with personalized language (first-person pronoun) are likely to be rumors.

*H*4: Information with unprofessional language (typographical errors) are likely to be rumors.

*H*5: Information with linkage language (hyperlinks) are likely to be rumors.

## Theoretical framework

Deception involves the manipulation of language and careful construction of messages or stories that appear truthful to others (20). Information Manipulation Theory (IMT) presents a multidimensional approach to comprehending deceptive messages by integrating Grice’s theory of conversational implicature with prior research on deception as information control (31–33). IMT utilizes Grice’s Cooperation Principle (CP) and its associated maxims as a conceptual framework to depict diverse forms of deceptive communication.

Within the IMT, deception is conceptualized as a result of covert violations of one or more of Grice’s four maxims: quality, quantity, relevance, and manner. Covert violations of the quality maxim involve the deliberate falsification or distortion of information. Violations of the quantity maxim can manifest as omission where pertinent information is intentionally withheld. Deception by evasion occurs when there are covert violations of the relevance maxim, redirecting attention away from pertinent information. Lastly, deception by equivocation arises from covert violations of the manner maxim, employing ambiguous or vague language to mislead.

By utilizing Grice’s CP and maxims, IMT offers a comprehensive framework for understanding the underlying mechanisms of deceptive communication. This approach enhances our comprehension of how individuals strategically manipulate information to influence others, shedding light on the intricate nature of deceptive messages across diverse contexts. This framework provides a valuable lens through which to examine deceptive communication and its role in social interaction. By identifying the various forms of deception and their underlying mechanisms, IMT informs our understanding of how individuals effectively control information to mislead others while highlighting the cognitive and social complexities involved in deceptive communication.

IMT provides a useful framework for understanding some aspects of human communication, but it cannot fully account for the complexity and richness of human communication. It has been criticized for not taking into account contextual and cultural factors (34–36). The tactics used by deceivers to manipulate information can vary depending on the cultural and contextual factors at play. For example, in some cultures, lack of first-person pronoun may be more effective in manipulating information, while in others, presence of first-person pronoun may be more effective. Additionally, the specific issues or topics being addressed can also impact the tactics used. It is important to consider these factors when analyzing the effectiveness of different strategies for manipulating information.

## Methods

### Data collection

We collected health rumors listed on two prominent platforms in China: Chinese Internet Rumor Clarification Platform^1^ run by Xinhua news and the Cyberspace Administration of China and Real-time Refutation of COVID-19 Rumors operated by Tencent News.^2^ The Report on the Development of China’s Internet Media (2022) indicates that these two websites dispelled most health rumors spreading in China, and they receive 200 million visits per year. Like Snopes.com, these two websites allow online users to submit rumors for other people to check out and verify. A team of professionals, researchers and reporters works closely together to set the record straight on each one of the rumors that has been collected on the websites by categorizing it as “true,” “false,” or “undetermined.” The websites have become the most authoritative platforms for debunking health rumors in the wake of the COVID-19 pandemic. There are few things that these websites do better than presenting rumor reports in their original form along with a veracity rating, facts, and detailed analysis of everything about that rumor.

A total of 1,903 health rumors were collected from the platforms in October 2022. The statements in all of the rumors were original. The veracity ratings provided by the platforms are assumed to be accurate. A total of 403 rumors were classified as “undetermined” and we thus excluded them from our final analysis of the data, leaving only 1,500 rumors to be analyzed.

### Data coding

The linguistic indicators associated with health rumors are presented in Table 3, whereas the coding schemes are described in Table 4. Two coders conducted the coding process in two distinct phases. During the initial phase, both coders independently analyzed a set of 300 randomly-selected rumors. Subsequently, they convened in-person to address and reconcile any divergences or conflicts that emerged during the coding process. The two coders demonstrated almost perfect inter-rater reliability for all measures as indicated by Cohen’s kappa (k; sentiment words: *k* = 0.94, *p* < 0.001; sensory words: *k* = 0.96, *p* < 0.001; continuous punctuations: *k* = 1.00, *p* < 0.001; extreme numbers: *k* = 0.99, *p* < 0.001; extreme adverbs: *k* = 0.98, *p* < 0.001; first-person pronoun: *k* = 1.00, *p* < 0.001; typographical errors: *k* = 0.98, *p* < 0.001; hyperlinks: *k* = 1.00, *p* < 0.001). In the second phase, they coded the remaining rumors that were assigned to them randomly.

**Table 3:** Examples of linguistic indicators in health rumors.

Categories	Indicators	Examples
Emotional language	Sentiment words (SentW)	高兴(happy), 悲伤(sad),恐怖(terrible)
	Sensory words (SensW)	温暖的(warm), 甜的(sweet), 冰冷的(cold), 硬硬的 (stiff)
	Continuous punctuations (ContP)	!!!!,???,!!??
Exaggerated language	Extreme number (ExtrN)	0.01, 999, 1.1, 99, 99.99, 100%, 一万 (ten thousand), 十亿 (billion)
	extreme Adverbs (ExtrA)	极其(extremely), 绝对(absolutely), 难以置信地(unbelievably), 完全 (totally), 唯一 (only), 最 (most)
Personalized language	First-person pronoun (FirsR)	我(I), 我们(we), 我的(my), 我们的(our)
Unprofessional language	Typographical errors (TypoE)	喝酒可以预防新冠酒精能杀四新冠病毒。(Drinking alcohol can prevent COVID-19, alcohol can kill the viruses.) It should be 杀死 (kill) not 杀四 (kill four).
Linkage language	Hyperlinks (Hyper)	钟南山院士空降北京。本该养老的年龄却不得不出山。https://www.cqcb.com/headline/2020-06-18/2557180_pc.html (Academician Zhong Nanshan arrived at Beijing. He should retire and enjoy his life, but he dedicated to fight again COVID-19.)

**Table 4:** Coding schemes for linguistic indicators of health rumors.

Variable	Coding scheme
Veracity	True information = 1, Rumors = 0
sentiment words	With = 1, without = 0
sensory words	With = 1, without = 0
continuous punctuations	With = 1, without = 0
extreme numbers	With = 1, without = 0
extreme adverbs	With = 1, without = 0
first-person pronoun	With = 1, without = 0
typographical errors	With = 1, without = 0
hyperlinks	With = 1, without = 0

### Data analysis

The research method used in this article is logistic regression. It is a statistical method used to analyze data and identify relationships between variables. In this study, logistic regression is employed to identify which features of health rumors are associated with their authenticity. Specifically, it examined how different linguistic indicators such as sentiment words, sensory words, continuous punctuations, extreme numbers, extreme adverbs, first-person pronoun, typographical errors as well as hyperlinks, are related to the probability that an item of information is rumor.

## Results

A sample of 1,500 items was examined, with 20% categorized as true information and 80% as rumors. Descriptive statistics are presented in Table 5. To explore the relationship between the dependent variables and the veracity of rumors, preliminary analyses were conducted.

**Table 5:** Descriptive statistics of variables.

		Full dataset (*n* = 1,500)		True information (*n* = 300)		Rumors (*n* = 1,200)	
	Variables	% (Value = 1)	% (Value = 0)	% (Value = 1)	% (Value = 0)	% (Value = 1)	% (Value = 0)
Emotional language	SentW	66.90 (1004)	33.10 (496)	29.00 (87)	71.00 (213)	76.40 (917)	23.60 (283)
	SensW	32.10 (481)	67.90 (1019)	40.70 (122)	59.30 (178)	29.90 (359)	70.10 (841)
	ContP	19.50 (293)	80.50 (1207)	11.70 (35)	88.30 (265)	21.50 (258)	78.50 (942)
Exaggerated language	ExtrN	42.90 (644)	57.10 (856)	30.30 (91)	69.70 (209)	46.10 (553)	53.9 (647)
	ExtrA	43.90 (658)	56.10 (842)	35.70 (107)	64.30 (193)	45.90 (551)	54.10 (649)
Personalized language	FirsR	16.00 (240)	84.00 (1260)	59.30 (178)	40.70 (122)	5.20 (62)	94.80 (1138)
Unprofessional language	TypoE	43.30 (649)	56.70 (851)	31.00 (93)	69.00 (207)	46.50 (558)	53.50 (642)
Linkage language	Hyper	27.90 (419)	72.10 (1081)	84.00 (48)	16.00 (252)	30.90 (371)	69.10 (829)

Separate Pearson’s chi-square tests revealed significant associations between the veracity of rumors and various factors. Specifically, we found significant associations between the veracity of rumors and the presence of sentiment words (*X^2^* = 241.67, *p* < 0.001), sensory words (*X^2^* = 12.24, *p* < 0.001), continuous punctuations (*X^2^* = 14.15, *p* < 0.001), extreme numbers (*X^2^* = 23.66, *p* < 0.001), extreme adverbs (*X^2^* = 9.83, *p* < 0.05), first-person pronoun (*X^2^* = 519.91, *p* < 0.001), typographical errors (*X^2^* = 24.90, *p* < 0.001) and hyperlinks (*X^2^* = 25.79, *p* < 0.001).

Nevertheless, the preliminary analyses conducted earlier were limited in their ability to examine all independent variables simultaneously. Therefore, to overcome this limitation and gain a comprehensive understanding of the relationship between the veracity of rumors and the eight independent variables, we employed logistic regression analysis.

Assumptions of logistic regression were meticulously assessed in line with established criteria. First, the response variable (veracity) was binary, adhering to the nature of logistic regression. Second, we ensured that observations were independent, thereby avoiding issues of dependency among the data points. Third, the absence of multicollinearity among the explanatory variables was confirmed through the examination of Variance Inflation Factor (VIF) values. The VIF values for the variables of interest yielded low values indicating no substantial multicollinearity (sentiment words: 1.03; sensory words: 1.01; continuous punctuations: 1.01; extreme numbers: 1.03; extreme adverbs:1.03; first-person pronoun:1.04; typographical errors: 1.02; hyperlinks: 1.01).

Moreover, we confirmed the absence of extreme outliers in our dataset, ensuring the robustness of our analysis. Furthermore, the determination of an adequate sample size was based on the number of independent variables. Given that eight independent variables were employed, we referred to existing guidelines (37) which recommend a minimum sample size of 500 to yield reliable and valid estimates. Thus, the chosen sample size was sufficient to draw meaningful conclusions from the logistic regression analysis performed.

Table 6 shows the findings of the logistic regression with all eight independent variables as predictors for the veracity of health rumors in China. The statistical significance of each predictor was corrected using the false discovery rate (FDR) with the Benjamini–Hochberg method. We found that the presence of sentiment words and continuous punctuations were both significantly related to the veracity. However, the presence of sensory words was not significantly related to the veracity. Thus, the finding partly support H1. The presence of extreme numbers and extreme adverbs were significantly related to the veracity of rumors, supporting H2. Moreover, the presence of first-person pronoun was significantly associated with the veracity, contradicting H3. In addition, typographical errors were significantly related to the veracity, supporting H4. Finally, hyperlinks were also significantly related to the veracity, supporting H5.

**Table 6:** Logistic regression findings.

*Predictors*	*Odds ratios*	*95% CI*	*p*	*FDR corrected p*
SentW	0.15	0.10–0.21	<0.001	<0.001
SensW	1.35	0.95–1.92	0.15	0.15
ContP	0.55	0.33–0.89	<0.05	<0.05
ExtrN	0.66	0.45–0.94	<0.05	<0.05
ExtrA	0.68	0.48–0.97	<0.05	<0.05
FirsR	20.27	13.89–30.01	<0.001	<0.001
TypoE	0.67	0.47–0.95	<0.05	<0.05
Hyper	0.58	0.38–0.87	<0.05	<0.05

*N* = 1,500, *R^2^* = 0.81.

## Discussion

Health rumors can have detrimental effects on individuals’ well-being and the overall public health. These rumors often circulate misinformation and false claims related to various health topics, which can lead to misinformation being widely spread and believed. Addressing health rumors requires effective communication strategies that involve providing accurate information, debunking misinformation, and promoting critical thinking skills. Identifying linguistic indicators to distinguish rumors and true information gradually becomes a promising approach (38, 39). Thus, our study aims to reveal linguistic indicators in terms of emotional, exaggerated, personalized, unprofessional and linkage language.

### Emotional language

Regarding the emotional language of rumors, our study found that the presence of sentiment words and continuous punctuation were significantly related to the veracity of rumors. However, the presence of sensory words was not a significant indicator. This finding partially supports hypothesis 1. Sensory words are processed more deeply and integrated more fully into memory than abstract words. Messages containing sensory details, such as smells or tastes, are rated as more believable and more likely to be shared. Previous studies have shown that liars tend to use fewer sensory words than truth-tellers (40, 41). Our study further suggests that the presence of sensory words is not a significant factor in distinguishing between health rumors and true health information. The interpretability and impact of sensory words can vary among individuals, making it challenging to establish a consistent relationship between the presence of sensory words and the veracity of health rumors. Additionally, our study revealed that rumors tend to employ more sentiment words than true information. This finding is consistent with previous studies that have found a positive and significant relationship between the use of emotional words and the dissemination of rumors (30, 42, 43). In terms of punctuation marks, a prior study found that unreliable articles contain more exclamation marks and less question marks in text (4). Our study further illuminated that the presence of continuous punctuations was a significant indicator to discern rumors and true information. Continuous punctuations, such as exclamation marks or question marks, can evoke emotional responses in receivers. These punctuations are often associated with emphasis or heightened emotional expression. When individuals encounter continuous punctuations in health rumors, it may trigger a stronger emotional response, such as surprise, excitement, or alarm. Emotional arousal can influence individuals’ perception and evaluation of information, potentially leading to an increased likelihood of believing and sharing rumors.

### Exaggerated language

With respect to exaggerated language of rumors, our study found that the presence of extreme numbers and adverbs were both significant indicators of rumors, which supports hypothesis 2. Zhang et al. (8) found that an item of information was more likely to be true if it contained elements such as numbers. However, this is not the case with extreme numbers. Other studies revealed that rumors containing extreme numbers spread faster and reached more users than those without them (44–46). Our study ascertained that extreme numbers was a significant indicator to distinguish health rumors and true health information. In terms of extreme adverbs, Rashkin et al. (47) found that the employment of action adverbs and manner adverbs can dramatize written text to attract readers’ attention. In addition, adverbs play a crucial role in shaping the overall persuasive effect of language. Our study revealed that extreme adverbs was a significant indicator to distinguish rumors and true information. The significance of extreme numbers and adverbs as indicators in discerning between rumors and true information may stem from two underlying factors. One possible explanation lies in the strategic behavior of rumor makers. Rumor creators may possess knowledge that messages containing extreme numbers and adverbs tend to be perceived as more credible and memorable. Therefore, they may deliberately include these linguistic elements in their rumors to make them more persuasive and increase their credibility. Moreover, rumor makers may employ extreme numbers and adverbs with the intention of creating an exaggeration effect. By utilizing these linguistic devices, they aim to capture and maintain the attention of readers, increasing the likelihood of successful persuasion and belief in the fabricated information spread through rumors.

### Personalized language

With respect to personalized language, our study suggested that rumors employed less first-person pronoun than true information, which contradicted hypothesis 3. There were two contradictory remarks concerning the first-person pronoun in previous lying studies. Some studies suggest that using first-person pronouns, such as referring to oneself using pronouns like “I” or “we” can indicate a higher level of self-involvement in order to establish a connection or empathy with the reader. As a result, liars may avoid using first-person pronouns to reduce this involvement (41, 48). While some studies indicated that deceivers tend to employ a higher frequency of first-person pronoun to enhance the credibility of their messages. This strategic use of first-person pronoun is believed to elicit a sense of immediacy and authenticity, which can be perceived by recipients as cues indicative of truthfulness (49). The findings of our study supported the notion that rumors exhibited a lower frequency of first-person pronoun compared to true information. This empirical evidence aligned with previous research, highlighting the tendency for deceptive information to employ fewer instances of first-person pronoun. This observation suggested that rumors may deliberately minimize the use of first-person pronoun as a strategic communication tactic. This behavior aims to distance the deceptive message from the individual, potentially reducing the perception of personal involvement and enhancing the perceived objectivity of the misinformation. In addition, Taylor et al. (49) proposed that deceptive individuals from individualistic cultures tend to use less first-person pronoun and more third-person reference, while individuals from collectivist cultures tend to use more first-person pronoun and less third-person reference. Our study revealed that rumors exhibited a lower occurrence of first-person pronoun in Chinese context. This finding highlights the significance of considering cultural factors in analyzing linguistic patterns in deceptive communication. It emphasizes the need for additional research on other contextual and individual variables that may influence the use of first-person pronoun in different cultural settings.

### Unprofessional language

With respect to unprofessional language, our study found that typographical error was a significant indicator to distinguish health rumors and true information, supporting hypothesis 4. Contrary to Sitaula et al. (19), our study had uncovered that rumors exhibited a greater prevalence of typographical errors. Nevertheless, it is noteworthy that these typographical errors do not impede the acceptance and dissemination of rumors. In the contemporary digital era, individuals are subjected to an incessant deluge of information, thereby encountering a condition of information overload. This phenomenon engenders a diminished level of attention and cognitive exertion during the assimilation of content. Consequently, typographical errors within rumors may be readily disregarded or accorded diminished significance in contrast to the overarching narrative of the rumor. This tendency is particularly pronounced when individuals are confronted with a copious volume of information, further accentuating the likelihood of typographical errors being overlooked.

### Linkage language

Previous studies have found that hyperlinks emerged as a mechanism for substantiating and proliferating the dissemination of rumors (8, 50). Our study further confirmed that hyperlinks could be a linguistic indicator to distinguish rumors and true information. Hyperlinks possess the potential to instill a cognitive bias known as the credibility illusion wherein individuals develop a perceived sense of trustworthiness and authenticity. This phenomenon is particularly pronounced when hyperlinks redirect users to sources or websites that exude an aura of authority and reliability. Given the entrenched tendency for individuals to assign undue credibility to information presented through hyperlinks, a noteworthy consequence arises: the failure to engage in critical evaluation of the accuracy and veracity of the information in question. Consequently, rumors spread unchecked due to the unintentional complicity of individuals who unknowingly contribute to the dissemination and propagation of misleading information.

## Conclusion

Online health rumors are prevalent and pose a great negative effect on health detection and disease prevention regulations and behaviors. Though much work is warranted to offer guidelines on evaluating online health information that could be easily employed by the general public, the identification of the authenticity of online health rumors has hardly received scholarly attention thus far. This study examined the linguistic indicators of the veracity online health rumors with real dataset in Chinese setting.

### Major findings

First, our findings suggest that certain linguistic indicators help to predict health rumors, which extends the literature on rumors. A few rules of thumb to be followed when judging online rumor veracity include assigning a priority to linguistic predictors such as sentiment words, continuous punctuations, extreme numbers, extreme adverbs, first-person pronoun, typographical errors and hyperlinks. Consistent with our findings, previous researches in field of deception revealed that significant differences existed between truthful messages and lying information in communication. However, our findings proposed that rumors had particular linguistic features.

This study represents a departure from previous research on rumors, which has primarily focused on enterprise- and government-oriented rumors, by examining the linguistic characteristics of health rumors. The findings suggest that rumormongers exhibit certain linguistic behaviors and tend to produce messages that lack detail and complexity. Furthermore, the study highlights the potential of leveraging language indicators to enhance individuals’ ability to differentiate between rumors and true information, thereby reducing the pernicious impact of rumors. Specifically, explicit warnings that draw attention to the linguistic differences between rumors and true information may prove effective in mitigating the negative effects of rumors.

Finally, this study utilized real dataset from a website for investigation as opposed to traditional methods such as surveys and experiments. On the one hand, it serves to remind online receivers that online health information should be taken with a grain of salt for the prevalence of rumors. This is especially significant because online receivers usually lack of careful attention and critical thinking on the health information they come across. On the other hand, it functions to remind health authority agencies an alternative method to verify and debunk rumors. Previous researches usually proposed that fact-checking was not enough to debunk rumors, profound actions beyond providing factual information should be taken. Analyzing and presenting the linguistic indicators in rumors could be a useful alternative to follow because it would finally improve the “self-defense” skills of online receivers against misleading health messages.

### Implications

The findings of our study yield both practical and theoretical implications. In terms of practical implications, the utilization of linguistic indicators for the identification and evaluation of credibility in online health information can facilitate individuals and organizations in making well-informed decisions regarding the reliability and trustworthiness of such information. By leveraging linguistic cues such as the presence of sentiment words, continuous punctuation marks, extreme numbers, adverbs, personalized language, typographical errors, and hyperlinks, individuals and organizations can enhance their ability to discern the authenticity of online health content. Consequently, this empowers us to engage in more critical evaluation and decision-making processes, thereby promoting a heightened understanding and assessment of online health information.

The absence of contextual and cultural considerations in IMT has long been subject to criticism (34–36). The theoretical implications of our findings reinforce the importance of incorporating cultural and contextual factors within the framework of IMT. By demonstrating the effectiveness of linguistic indicators in identifying and assessing the credibility of online health information, our study highlights the need to account for the influence of cultural and contextual variables in understanding the manipulation of information. The findings indicate that individuals who deceive others in various contexts and cultures may utilize different strategies to manipulate information. For instance, Taylor et al. (49) demonstrated that individuals from collectivist cultures exhibit a higher frequency of first-person pronoun usage and a lower frequency of third-person reference in deception studies. However, our findings contradict this trend, as we observed that health rumors in collectivist cultures, such as China, contained a lower frequency of first-person pronoun usage. This underscores the necessity of expanding information manipulation theory to encompass a more comprehensive understanding of how cultural and contextual factors shape the manipulation of information in various contexts.

## Limitations

Despite its contribution, this study should be viewed in light of three limitations that could be addressed in future studies. First, the present study drew upon a sample of authentic rumor data obtained from two reputable Chinese counter-rumor websites, wherein 80% of the data pertained to rumors. While the employment of logistic regression analysis was deemed appropriate for the purposes of the study, it is recommended that future research endeavors extend data collection efforts to encompass a broader range of websites. Second, this study extracted and analyzed eight linguistic indicators, but it mainly focused on lexical-level linguistic properties. Future research could extend to cover semantic-level and pragmatic-level linguistic properties. Second, this study took a synchronic investigation of online health rumors and revealed a series of significant linguistic predictors. However, rumors could be dynamic in spreading process, which enables various rumormongers to edit and reedit the messages. Future studies could take a diachronic perspective to investigate the changes and development of linguistic properties of health rumors.

## Data availability statement

The raw data supporting the conclusions of this article will be made available by the authors, without undue reservation.

## Author contributions

JZ: Conceptualization, Data analysis, Writing – original draft, Writing – review & editing. CF: Conceptualization, Writing – original draft, Writing – review & editing, Methodology, Supervision.

## Footnotes

^1^ http://www.piyao.org.cn/

^2^ https://vp.fact.qq.com/
